# The Idea of the Surgical "At Home."

**Published:** 1913-04-12

**Authors:** 


### The Hospital

The Workers' Newspaper of Administrative Medicine and Institutional Life, Administration, National Insurance and Health.

No. 1397, Vol. LIV. SATURDAY, APRIL 12, 1913. PRICE ONE PENNY.

### THE IDEA OF THE SURGICAL "AT HOME."

There are few communities in which there is such freedom of mutual criticism and appreciation as there is in the teaching hospitals of this country. Every member of the staff, while he is in the wards, or operating theatres, or out-patient departments, is perpetually in a light as fierce as that which is said to beat about a throne. He can do, or leave undone, nothing whatever which is not open to the gaze of every passing student; and though his "firm" of house officer and dressers (or clerks) will be strictly loyal to him in many ways, they do not consider themselves under any obligation to refrain from frank and free comments to their fellows upon anything he does. So much is this the case, that more than one well-known and highly-venerated teacher has made a stock witticism of the inevitableness of such criticism from his pupils. Nor is such a custom at all one to be deplored, so long as it is kept within such bounds as any gentleman will know how to impose upon himself. Rather is it an advantage, for it acts as a constant spur to the senior to keep abreast of the latest advances, and so raises the general standard perhaps a shade higher than it otherwise might be. Another result is that an exceptionally brilliant man may, and often does, impress some of his outstanding characteristics upon the whole of his school, for he acts as a standard which public opinion forces all the others to strive after also. Thus some particular feature in surgical technique, or some favourite prescription, comes in time to be almost universal at one hospital, whereas at another it may be almost unknown.

Members of the medical staff of teaching hospitals, when on a holiday, not infrequently visit famous clinics abroad for the purpose of seeing what is being done and how things are being done elsewhere. But they very seldom visit the other schools in their own district, partly from diffidence, partly from lack of time, partly because the custom has not hitherto existed. Yet, on the face of it, they have much to gain by such intercourse, whereby the special merits of some well-established practice at one hospital may be disseminated also throughout others. Fortunately, in London steps have lately been taken to bring about such a habit. A committee was appointed some little time ago, and it has been decided that once a year each hospital shall have an "At Home," to which the staffs of the other teaching hospitals shall be invited. So far, it is only the surgeons who have adopted this happy innovation; and the special feature of any surgical reception is, naturally, operative work.

The first "At Home" was held in February at St. Bartholomew's Hospital, priority being accorded to that foundation because of its seniority in point of years. At a convenient hour four of the surgeons each began an operation; the other four members of the surgical staff took a similar turn later on. Members of the staff acted as stewards, and tea was served afterwards in the Staff Common Room. All the visitors were given the best possible chances of seeing what was going on. Eighty invitations were issued, and thirty surgeons attended, including at least one representative from each of the teaching hospitals. In due order of precedence the other hospitals will "receive" in the same way; and the list is interesting also as a reminder of the dates at which the hospitals were founded. St. Bartholomew's is getting near its eighth completed century, having been founded in 1123. St. Thomas's stands next, 1223, and was allotted March. This month, April, it is Westminster Hospital's turn (1719). In May Guy's has the honour (1721); in June it will be St. George's (1734). The London is "At Home" in July (1740); and Middlesex in October (1745). Charing Cross (November) was founded in 1822; University College (December) in 1826; King's College (January) in 1829; and St. Mary's (February) in 1850.

The scheme thus auspiciously initiated is one of those things which are so simple when once some ingenious person has thought of them. We do not know, in the present case, whose the inventive mind is; but we can congratulate him impersonally. The custom, once established, will soon take root as part of the natural order of things; and in a little while everyone will wonder why it was never done before. Nor do we see any necessity why it need stop at the teaching general hospitals. At the special hospitals, especially at some of them which are noted for the original work which has been done in them (for instance, St. Peter's, St. Mark's, and the Queen Square Hospital for Paralysis and Epilepsy), the general surgeon would have opportunities of seeing practised the latest devices and improvements, which otherwise he can only read of and may overlook altogether. There is no reason, at this time of day, why any hospital surgeon should take it upon himself to explore a kidney—let us say—without having first used a cystoscope, had an *x*-ray plate made, catheterised the ureters, and generally given the patient the advantage of the last ten years' research in this branch of surgery. Yet it is still done in London, we regret to say. Because this new idea tends to destroy parochialism and to level up, we welcome it with cordiality and good wishes.

